# Targeted inhibition of long non-coding RNA H19 blocks anaplastic thyroid carcinoma growth and metastasis

**DOI:** 10.1080/21655979.2019.1642722

**Published:** 2019-07-19

**Authors:** Honglai Zhang, Ying Yu, Kejun Zhang, Xinfeng Liu, Yan Dai, Xuelong Jiao

**Affiliations:** a Department of Thyroid Surgery, The Affiliated Hospital of Qingdao University, Qingdao, Shandong, China; b Department of Nuclear Medicine, The Affiliated Hospital of Qingdao University, Qingdao, Shandong, China; c Department of Endocrinology, Linyi Central Hospital, Linyi, Shandong, China; d Department of General Surgery, The Affiliated Hospital of Qingdao University, Qingdao, Shandong, China

**Keywords:** Anaplastic thyroid carcinoma, invasion, tumorigenesis, molecular therapy, long non-coding RNA H19

## Abstract

Long non-coding RNA H19 (H19) is highly expressed in cancers and is considered to highly correlate with the extent of malignant degree. The present study was performed to determine the expression levels of H19 in anaplastic thyroid carcinoma (ATC) tissues and the role of H19 in ATC 8505C cells *in vitro* and *in vivo*. Expression of H19 was detected in 19 ATC and 19 normal thyroid tissues by real-time quantitative polymerase chain reaction. Utilizing the siRNA or short hairpin RNA (shRNA) directed against human H19 (H19 siRNA or shRNA H19) depleted H19 in ATC 8505C cells and characterized the outcomes. The results showed that H19 was overexpressed in ATC tissues. Targeting H19 inhibited proliferation, migration, and invasion and induced apoptosis in 8505C cells *in vitro* and inhibited tumorigenesis and metastasis *in vivo*. Therefore, the H19 might be an effective target for ATC molecular therapy.

## Graphical Abstract

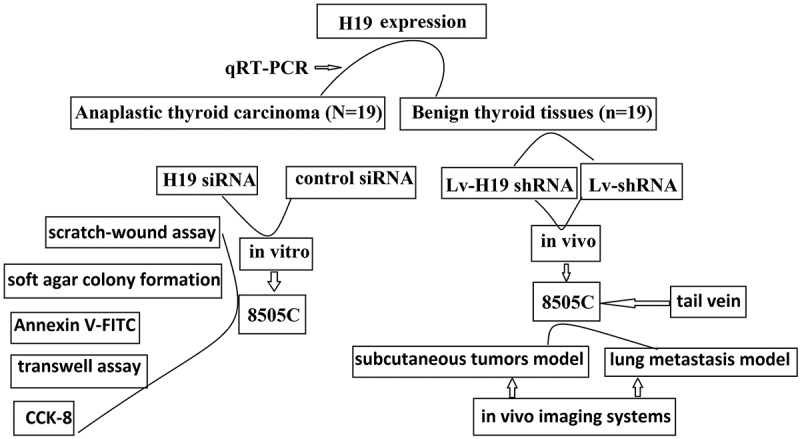

## Introduction

Anaplastic thyroid carcinoma (ATC), with a mean survival rate of 4–12 months after diagnosis, is a rare tumor that accounts for 2–5% of all thyroid cancers. Clinical evident indicatedthat 50% of ATC patients have had distant metastases at the time of clinical diagnosis, as to the ATC patients who have not had distant metastasis, they will also be classified as stage IV by the American Joint Committee on Cancer. ATC is not sensitive to any current systemic treatment, including chemotherapy, radiotherapy, and radioiodine (^131^I) therapy or thyroid-stimulating hormone (TSH) inhibition. Although several promising targeted therapies [1–3] and multimodal therapy strategies have been explored [4–6], there is no definitive evidence to date that these patients can prolong the survival time. Obviously, new treatments are urgently needed. Waiting for the advent of new genome-wide approaches, the analysis of the molecular mechanisms involved in the pathogenesis of ATC still remains the only available tool for planning any targeted therapy.

Long non-coding (Lnc) RNAs are defined as non-coding transcripts that are more than 200 nucleotides in length. According to regulatory mechanisms on the expression of target genes or on other molecules, lncRNAs can act as signals (e.g., *Xist*), decoys (e.g., *Gas5, MALAT1*), scaffolds (e.g., *NEAT1*), guides (e.g., *HOTAIR, linc-p21*), competing endogenous RNAs (ceRNAs) (e.g., *linc-MD1*) [7], and others. Their expression deregulation plays an important role in the progress of cancer. The long non-coding RNA (lncRNA) H19 can function either as a tumor promoter or as a tumor suppressor, depending on the type of cancer, development stage, or molecular background [8–11]. The H19 was upregulated in many tumors including esophageal squamous cell carcinoma [12], lung cancer [13], gastric cancer [14], and breast cancer [15], and H19 overexpression contributes to poor prognosis in these patients. However, in osteosarcoma [16] and renal cell carcinoma [17], H19 overexpression contributes to a good prognosis. Thus, it is possible that H19 may be capable of totally opposite functions, with the specific functions being largely dependent on cellular context and partners.

Liang et al. reported that H19 was overexpressed in papillary thyroid carcinoma (PTC), and H19 overexpression contributes to epithelial–mesenchymal transition (EMT) [18]. However, Wang et al. reported that H19 overexpression inhibited invasion and EMT in PTC cells [19], and Lan et al. reported that H19 downregulation contributes to proliferation and migration in PTC cells [20]. H19 expression and its role in ATC were still unclear.

The present study profiled the expression of H19 in 19 ATC and 19 benign thyroid nodes via quantitative reverse-transcriptase polymerase chain reaction (qRT-PCR) analysis. We further determined whether H19 could act as the gene therapy target for ATC *in vitro* and *in vivo*. Our study demonstrates that H19 is overexpressed in ATC tissues. Targeting H19 expression might inhibit tumorigenesis and metastatic ability of ATC cells. The H19 gene might be an effective target for tumor molecular therapy.

## Materials and methods

### ATC specimens

Specimens were collected from our frozen tumor tissue bank including 19 frozen ATC tissue and 19 patients with benign thyroid nodes who underwent thyroidectomy and/or or fine needle aspiration (FNA) tissues at the affiliated hospital of Qingdao University between January 2012 and December 2018.

### Cell culture

The human ATC lines 8505C, SW1736, KAT18, and Cal-62 were obtained from German Collection of Microorganisms and Cell Culture (DSMZ, Braunschweig, Germany) and CLS Cell Lines Service GmbH (Germany) and kept in our laboratory. Nthy-ori 3-1 primary human thyroid follicular epithelial cells were purchased from ATCC, Shanghai, China. All the ATC cell lines were also authenticated using short tandem repeat (STR) analysis as described in 2012 in ANSI Standard (ANSI/ATCC ASN-0002-2011 Authentication of Human Cell Lines: Standardization of STR Profiling) by the ATCC Standards Development Organization and negative for mycoplasma contamination, carried out by Guangdong Hybribio Biotech Ltd. The ATC cells were routinely cultured in DMEM/F12 (Thermo Fisher Scientific, CA, USA) and Nthy-ori 3-1 were grown in RPMI 1640 (Life Technologies, Naerum, Denmark) medium, supplemented with 10% fetal bovine serum (Thermo Fisher Scientific) and penicillin (100 U/ml) and streptomycin (100 μg/ml) (Thermo Fisher Scientific), and maintained in a humidified 5% CO_2_ atmosphere at 37°C.

### siRNA transfection

Validated H19-specific and non-silencing siRNA (control siRNA) were purchased from Santa Cruz Biotechnology (Santa Cruz, CA, USA). For *in vitro* transfections, 2 μl of 100 μM or a total of 2 μg H19 siRNA and 3 μl Lipofectamine 2000 (Invitrogen) were each first diluted in 100 μl of serum-free media and then mixed and incubated for 30 min. The mixture was added to 5 × 10^5^ 8505C cells in a six-well plate in serum-free media (1 ml) and incubated for 6 h. The final concentration of the siRNA was 125.3 nM. Transfected cells were maintained for 24–72 h in complete media. All experiments were conducted in triplicates, and at least three independent experiments were performed.

### Lentivirus production and infection

Short hairpin RNA (shRNA) directed against human H19 or scrambled oligonucleotides were ligated into the LV-3 (pGLVH1/GFP + Puro) vector (GenePharma, Shanghai, China). The viruses were packaged in HEK293T cells according to standard protocols, and the virus particles were harvested 72 h later. The packaged lentiviruses were named Lv-H19 shRNA and Lv-scramble. 8505C cells were transfected with Lv-H19 shRNA and Lv-scramble for 48 h using Lipofectamine 2000 following the manufacturer’s instructions. In order to select the stably transfected cells, after 48 h transfection, the cells were treated with puromycin (2 μg/ml) for 2 weeks.

### RNA extraction and qRT-PCR analyses

Total RNA was isolated using the TRIZOL reagent (Invitrogen, Shanghai, China) from tissues and cultured cells according to the manufacturer’s protocol. The RNA quality was determined on an Agilent 2100 Bioanalyzer (Agilent Technologies, Guangzhou, China) using the RNA 6000 Nano Assay according to the manufacturer’s protocol. The purity of RNA was assessed by the 260/280 and 260/230 ratios of absorbance values using a Nanodrop ND-1000 spectrophotometer. The isolated RNA was reverse transcribed to cDNA using a Reverse Transcription Kit (Takara, Dalian, China). For qRT-PCR, 2 μl of cDNA was added to Power SYBR Green (Takara). The results were normalized to the expression of GAPDH. Relative fold change in H19 gene expression was calculated using 2^−ΔΔCt^ method and normalized with respective controls. The primers used in our study were as below: The primers used for H19 were 3ʹ-TACAACCACTGCACTACCTG-5ʹ, 5ʹ-3ʹTGGCCATGAAGATGGAGTCG; GAPDH forward 5′-GCACCGTCAAGGCTG AGAAC-3′ and reverse 5′-TGGTGAAGACGCCAGTGGA-3′.

### Proliferation analysis

The cell proliferation was detected using cells counting kit 8 (CCK-8). Modified cells and negative cells were seeded into 96-well plates (100 μl/well; 2500 cells/well) and 4 wells in each group. Then, the cells were incubated at 37°C in an environment with 5% CO_2_. At 24, 48, and 72 h, 10 μl of CCK-8 solution (cat. No. CK04, Dojindo Co., Ltd., Japan) was added into each well, followed by incubation for 3 h, and the solution color was monitored. When the difference in the color was significant between groups, absorbance was measured at 450 nm. The experiment was performed three times.

### Apoptosis analysis

8505C cells transfected with Lv-shRNA or Lv-scramble for 72 h were collected. Cell apoptosis was detected using the Annexin V-FITC Apoptosis Detection kit (Sigma–Aldrich, St. Louis, MO, USA) according to the manufacturer’s instructions. The percentage of apoptotic cells was defined as the sum of early apoptosis (AnnexinV-positive) and late apoptosis (Annexin V-positive and Propidium Iodide(PI)-positive) cells.

### Colony formation assay

The soft agar colony formation assay was used to determine the carcinogenesis and malignant transformation *in vitro*. The stable Lv-shRNA or Lv-scramble transfected 8505C cells (10^5^ cells/well) were mixed with 0.3% agar that dissolved in the growth medium and plated on top of a bottom layer of 0.7% agar that dissolved in the growth medium, in a six-well plate. A layer of completed medium, approximately 200 μl, were maintained over the upper layer of agar and changed medium every 3 days. After 21 days, the colonies were dyed with Cristal Violet (0.01% solution) and washed with phosphate buffer saline (PBS). Images of clones were obtained using an inverted phase-contrast microscope (Olympus Corporation) equipped with a digital camera. The number of clones was determined using Image-Pro Plus software version 6.0.

### Cell migration assay

The cell migration was detected using scratch-wound assay. Briefly, 90% confluent cell layer was scratched with a 10 μl pipette tip, and detached cells were removed by washing with PBS. The cells were cultured in medium with 2% fetal bovine serum (FBS) for 48 h. Images of the scratch wounds were obtained using an inverted phase-contrast microscope (Olympus Corporation) equipped with a digital camera at 0, 24, and 48 h. The experiment was performed three times. The wound width was determined using Image-Pro Plus software version 6.0.

### Cell invasion assay

The cell invasion was detected using transwell assay with Corning^R^ BioCoat^TM^ Matrigel^R^ Invasion Chamber (cat. No. 354480, Corning, NY, USA). Cells were seeded into transwell upper chamber at a density of 5 × 10^4^ cells/well in 200 μl serum-free medium with three replicates, and 600 μl medium with 10% FBS was added to the lower chambers. After incubation for 24 h, the non-invasive cells on the upper membrane of the insert were erased by a cotton swab. The migration cells adhered to the lower membrane surface were fixed with 4% paraformaldehyde for 30 min at room temperature (RT), stained with crystal violet for 30 min, and then the number of cells was counted under a microscope in five random optical fields. The experiment was performed three times.

### *Analysis of the tumorigenesis and metastasis of modified cells* in vivo

All animal studies were approved by the Animal Ethical and Welfare Committee of the affiliated hospital of Qingdao University. Cell tumorigenesis and transplant were detected using *in vivo* imaging systems (IVIS, Xenogen Corp., CA, USA). The stable Lv-shRNA or Lv-scramble transfected 8505C cells (1 × 10^7^ in 100 μl PBS) were injected into the 4- to 6-week-old female nude mice (BABL/c background) through the subcutaneous or tail vein. The 18 mice were randomly allocated into one of the six treatment groups and housed in Specific Pathogen Free (SPF) room with free access to food and water. To evaluate the tumor growth condition, the mice carrying v-shRNA or Lv-scramble cells were intraperitoneally administered with 150 mg/kg body weight D-luciferin, and the cells emitted a visual light signal that can be monitored using IVIS Spectrum. The photon flux from the mouse is proportional to the number of light-emitting cells, and the signal can be measured to monitor tumorigenesis and metastasis.

### Statistical analysis

Data were presented as mean ± standard deviation (SD) from at least three separate experiments. The statistical significance of the differences between groups was assessed using *t*-test. Statistical analysis was performed using SPSS software version 13.0 (IBM Corp., Armonk, NY, USA). *p* < 0.05 was considered a statistically significant difference.

## Results

### Expression of H19 in ATC tissues and ATC cell lines

Of the 19 ATC tissues, 5 tissues were downregulated and 14 ATC tissues were upregulated compared to Benign thyroid nodules (BTNs). The mean H19 expression was 4.356 ± 1.07 in ATC tissues and 2.7257 ± 0.876 in BTN tissues (*p* = 0.006). H19 expression was 0.436 ± 0.12 in the Nthy-ori 3-1 cells and 3.172 ± 0.86, 1.282 ± 0.29, 0.845 ± 0.16, and 2.575 ± 0.65 in 8505C, SW1736, KAT18, and Cal-62 cells, respectively. H19 expression was significantly increased in ATC cell lines compared to the Nthy-ori 3-1 primary human thyroid follicular epithelial cells. Highest expression of H19 was found in 8505C cells, so we used 8505C cells for further study.

### Lv-H19 shRNA inhibits H19 expression in 8505C cells

8505C cells were transfected with Lv-H19 shRNA or Lv-scramble for 48 h. Lv-H19 shRNA significantly decreased H19 expression in 8505C cells (0.53 ± 0.12), compared with untreated (3.172 ± 0.86) or Lv-scramble treated (2.986 ± 0.87) 8505C cells (*p* < 0.001). Expression of H19 was also significantly decreased in the Lv-H19 shRNA stably transfected 8505C cells (0.14 ± 0.08) compared to the Lv-scramble stably transfected 8505C cells (2.912 ± 0.86) (*p* < 0.001). These data indicated that Lv-H19 shRNA was successfully transfected into the 8505C cells.

### *Targeting H19-inhibited proliferation, colony formation, and induced apoptosis in 8505C cells* in vitro

**Figure 1.**
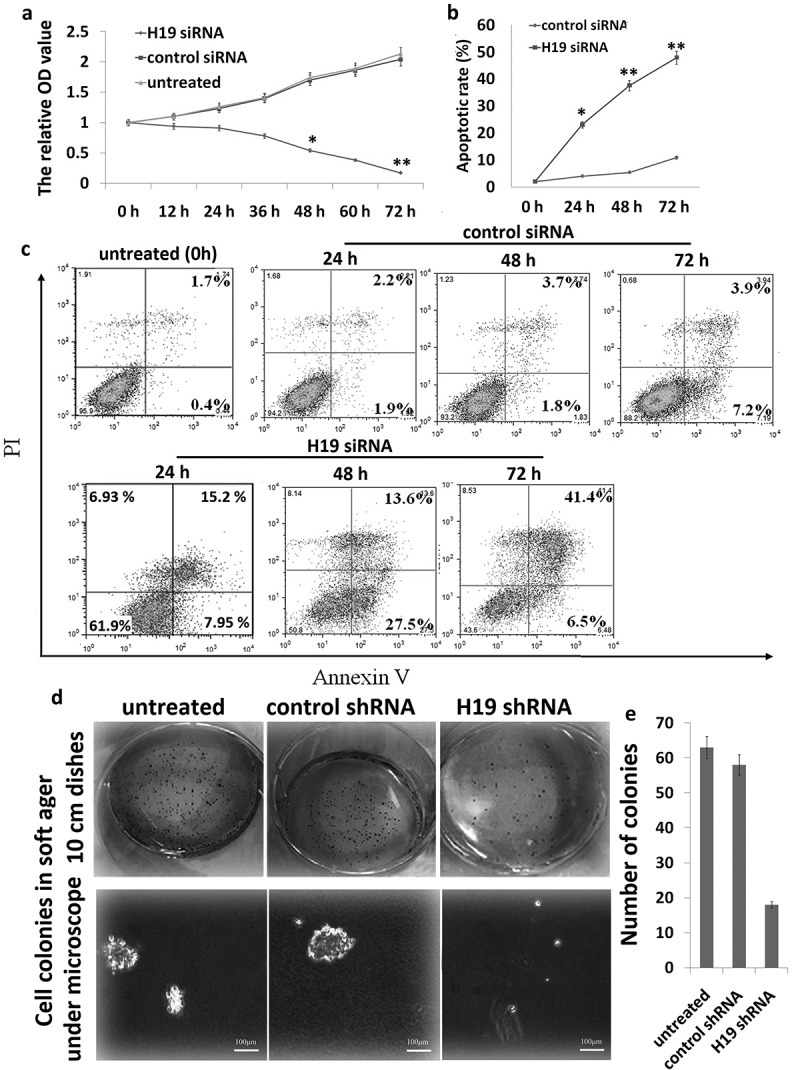
Targeting H19 in 8505C cells *in vitro*. (a) Cell index curve of proliferation through cell counting 8505C. (b,c) The apoptotic rate of 8505C cells was detected by FITC-annexin V labeling and flow cytometric analysis; (d) colony morphology; (e) efficiency of colony formation. *p < 0.05; **p < 0.01 compared with the control group.

8505C cells were transfected with Lv-H19 shRNA or Lv-scramble for 0–72 h. When compared with Lv-scramble, Lv-H19 shRNA revealed a significantly declined trend in their proliferation after 72 h (Figure 1(a)), indicating targeting H19 significantly inhibited 8505C cell proliferation. Flow cytometric assays showed that apoptotic cells were significantly increased in the Lv-H19 shRNA transfected 8505C cells compared to the Lv-scramble transfected 8505C cells (Figure 1(b,c)). Clonogenic proliferation assays also showed that Lv-H19 shRNA significantly inhibited cell growth in the 8505C cells (Figure 1(d,e)).

### Targeting H19-inhibited 8505C cell migration

**Figure 2.**
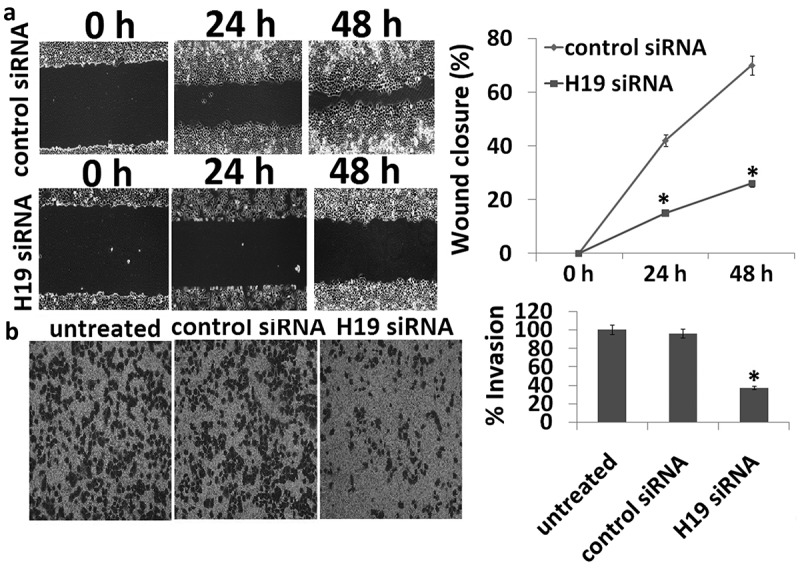
Scratch wound and transwell migration assay. (a) The wound healing ability for the transfectants was measured by wound healing assay. The control siRNA had a higher wound healing ability than that of H19 siRNA. (b) Representative bright-field images of the cell invasion (bar = 100 μm) and quantification of invasive cells at 24 h are shown. Data are presented as mean ± SD. *p < 0.05.

The migration of targeting H19 in the 8505C cells was determined through wound scratch-wound assay. For quantification of migration ratio, three images were captured and measured the width of wound at the same site on 0, 24, and 48 h, with three repeats and three times. When compared with control siRNA, the cell migration ability was inhibited in H19 siRNA transfected 8505C cells (Figure 2(a)).

### Targeting H19-inhibited 8505C cell invasion

The invasion of targeting H19 in the 8505C cells was determined through transwell assay. For quantification of invasion cells, three images were captured for cell count at the outer bottom of the inner chamber on 24 h, with three repeats and three times. When compared with control siRNA, the cell invasion ability was inhibited in H19 siRNA transfected 8505C cells (Figure 2(b)).

### *Targeting H19-inhibited tumorigenesis and metastasis* in vivo

**Figure 3.**
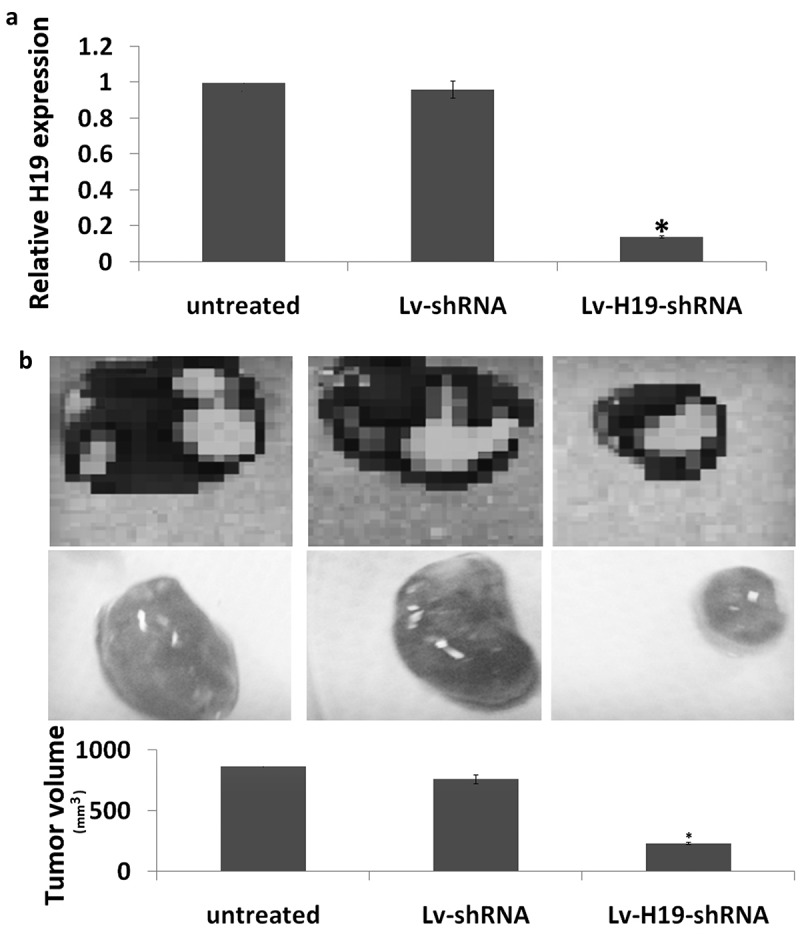
Targeting H19 on tumorigenesis *in vivo*. (a) Expression of H19 in subcutaneous tumorigenesis by qRT-PCR assay. (b) Tumor volume at 4 weeks after the nude mice were injected stable Lv-shRNA or Lv-scramble transfected 8505C cells (1 × 10^7^ in 100 μl PBS) through subcutaneous. **p < 0.01.

The tumorigenesis of targeting H19 was determined through subcutaneous tumorigenesis. As shown in Figure 3(a), H19 expression was significantly decreased in the Lv-H19 shRNA transfected subcutaneous tumors compared to the Lv-scramble transfected subcutaneous tumors. Furthermore, targeting H19 significantly inhibited the subcutaneous tumorigenesis *in vivo* (Figure 3(b)).

**Figure 4.**
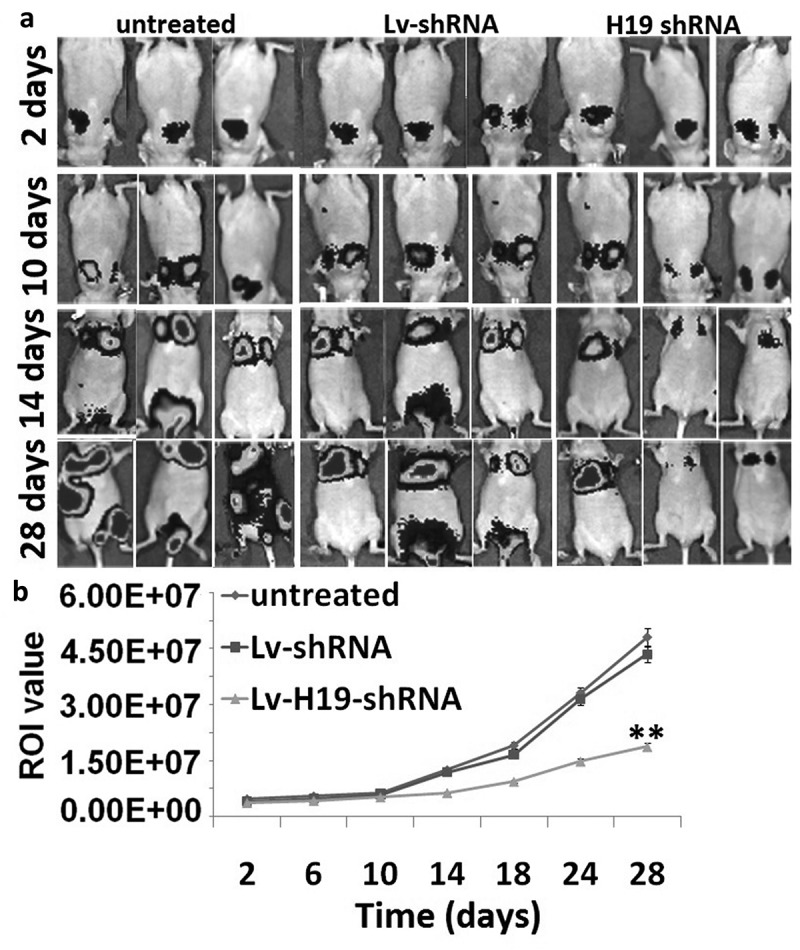
Targeting H19 on tumor metastasis *in vivo*. (a) The tumor metastasis after tail vein injection of 8505C/H19 shRNA *in vivo*. (b) The region of interest (ROI) analysis of the whole body based on tumor metastasis.**p < 0.01.

After tail vein injection, the signals produced from 8505C-Fluc2 cells mainly located in the lungs from 2 to 10 days and the luciferase signals of 8505C-Fluc2 cells from groups showed insignificant difference (Figure 4). After 10 days, untreated and Lv-scramble groups had transferred to the location of liver and kidney. From 14 days to experimental terminal, the luciferase signals of the whole body from group of untreated and Lv-scramble had a higher level, and the Lv-H19 shRNA groups were with lower luciferase signal level. Therefore, H19 downregulation inhibits tumor metastasis *in vivo*.

## Discussion

ATC is a devastating disease and has no effective treatment options. Understanding the specific mechanisms involved in the deregulation of cell growth, migration, and invasion of ATC is necessary for developing new therapies. Targeted molecular therapy has improved morbidity and mortality in a number of cancers.

LncRNA-H19 has been identified as one cancer-related lncRNAs. The high expression level of H19 was considered to be correlated with diverse human disorders and cancers. Growing evidence showed that this H19 gene-encoded 2.3 kb lncRNA functioned in tumorigenesis and cancer progression. The development of cancer may be promoted by the upregulated expression of H19 [21]. Little is known about the role of H19 in ATC. In breast cancer, H19 was associated with stem cell phenotype in ALDH1-positive breast cancer; furthermore, H19 regulates breast cancer stem cells (CSC) and is associated with poor prognosis in breast cancer patients, particularly in triple-negative subtype [15]. In lung adenocarcinoma, highly expressed H19 could serve as predictors for the poor prognosis [22]. H19 is also shown to be overexpressed in gastric cancer tissues, with increased expression of H19 relating to advanced pathological tumor stage and pathological tumor node metastasis stage [23]. In PTC tissues, H19 was significantly higher than paired paracancerous tissue or normal tissues, and overexpression of H19 was correlated with higher tumor burden of PTCs [18]. Li et al. also found that H19 is highly expressed in papillary thyroid cancer stem cells (PTCSCs) and PTC tissue specimens, which is correlated with poor overall survival [24]. However, Lan et al. have reported that H19 is downregulated in PTC tissues and in PTC cell lines compared to controls, and decreased H19 expression was correlated with lymph node metastasis [20]. Wächter et al. have recently reported 12 ATC patients, of which 6 ATC samples showed an overexpression of H19, 2 of them a downregulation, and 4 showed a stable expression in comparison with normal thyroid tissue used as control [25]. Of the 19 ATC tissues in our study, 5 tissues were downregulated and 14 ATC tissues were upregulated. The mean H19 was increased in 19 frozen ATC tissues compared to the 19 patients with benign thyroid nodes who underwent thyroidectomy and/or or FNA tissues. Our study was agreed to Li’s and Wächter’s [24,25] but contrary to the Lan’s [20]. The expression level and role of H19 in different tissues are different. Even in thyroid cancer tissues, the results of different authors are different. In my opinion, these controversial results in PTCs or ATCs might be because of radiotherapy in some patients. Whether radioiodine or radiotherapy affects H19 expression needs further investigation.

Numerous studies have shown that H19 can be used as a potential tumor marker for targeted therapy of malignant tumors, including PTCs. To investigate the potential effects of targeting H19 on ATC, an H19-interfering siRNA oligonucleotide was constructed and transfected into the ATC 8505C cells, which has rich H19 expression. Our results indicated that targeting H19 by siRNA significantly decreased H19 expression in the 8505C cells. Furthermore, targeting H19 significantly decreased proliferation and invasion and increased 8505C cell apoptosis.

Distant metastasis to the lung represents one of the most lethal characteristics of thyroid cancer, and there are currently no effective therapeutic options for these patients. Because of the role of H19 in lung metastases in other tumor types, and the importance of distant metastases in thyroid cancer, we developed an experimental metastasis model using an injection approach in a murine model of ATC to study tumor growth and metastasis. The use of bioluminescence is well established in models for metastasis and tumor growth in other *in vivo* tumor models such as mammary and colon cancer [26,27]. Monitoring tumors by bioluminescence measurements permitted early randomization of mice. Although a number of confounding factors, such as tumor necrosis and alterations in tumor vascularity caused by a targeted agent, may affect tumor bioluminescence over time, our bioluminescence measurements corresponded well with tumor volumes at the time of necropsy. In our study, we could validate this model *in vivo* by showing that targeting H19 using shRNA reduced H19 expression level, strongly reduced tumor burden, and abolished metastasization in a thyroid orthotopic ATC mouse model. Using this approach, targeting H19 significantly inhibited metastatic tumor progression by approximately eightfold.

It has reported that many ATCs are derived from malignant degeneration of preexisting well-differentiated PTCs, and ATC is a phenomenon that occurs late in the development of PTCs [28]. Dey et al. have found that in the embryonic period, H19 mostly promotes differentiation, while in the adult, it is rarely expressed in noncancerous tissues and has tumorigenic properties, and induction of H19 at later stages may even worsen the tumorigenic phenotype [29]. This may be the mechanisms why H19 is the tumor suppressor in PTC and promoter in ATCs.

## Conclusion

These results provide evidence that H19 was upregulated in ATC, and H19 siRNA (shRNA) has on-target effects and thus validates that H19 has an important role in both antitumor and antimetastatic role and may be a potential therapeutic target for the treatment of ATCs.

## Research highlights

1. H19 is upregulated in anaplastic thyroid carcinoma.
2. Targeting H19 inhibits proliferation, migration and invasion, and tumorigenesis in 8505C cells *in vitro.*
3. Targeting H19 inhibits tumorigenesis and lung metastasis in 8505C cells *in vivo.*
4. H19 may be a potential therapeutic target for the treatment of ATC.
